# Post-steroid rebound in COVID-19 pneumonitis: a case series and review of the literature

**DOI:** 10.1186/s12890-025-03749-z

**Published:** 2025-09-30

**Authors:** Numbere K. Numbere

**Affiliations:** Worthing Hospital, University Hospitals Sussex NHS Foundation Trust, Worthing, United Kingdom

**Keywords:** Post-steroid, COVID-19 pneumonitis, Rebound COVID-19, Steroid wean, Post COVID-19 pneumonitis

## Abstract

We report a retrospective case series of COVID-19 pneumonitis (C19P) patients in hypoxic respiratory failure who experienced a symptom rebound upon cessation or weaning of steroids following an initial positive response. The post-steroid rebound phenomenon in C19P is not well described in the literature and we aim to add to the body of evidence exploring this pathology.

**Methods**: Post-steroid rebound COVID-19 pneumonitis (PSRCP) cases at our institution were identified for notes review from respiratory department follow-up records. The inclusion criteria were as follows:

1. Hospital admissions with radiologically and PCR-confirmed C19P.
2. Administration of a corticosteroid course for the indication of hypoxia due to C19P.
3. An objective relapse of the index presentation with differential diagnoses other than post-steroid rebound excluded by appropriate clinicians.

A literature search was performed using Medline, Ovid and Google Scholar and the search terms “rebound and COVID-19”, “rebound and COVID-19 and pneumonitis” “post-COVID and pneumonitis” “relapse and COVID-19”, “relapse and coronavirus and pneumonitis”.

**Results**: Eighteen patients were identified between 2021 and 2024 with ages ranging from 48 to 80 years. The most common comorbidities were hypertension (50%) and obesity (39%) while 89% had a history of regular smoking. Seventeen of the 18 had evidence of hyperinflammation at first C19P presentation with a C-reactive protein (CRP) ≥ 75 mg/dl. Notably, 15 patients had a CRP blood test at least 48 h prior to discharge, steroid cessation or weaning and of these, 11 (73%) showed persisting CRP elevation. Seventeen of the 18 responded upon diagnosis of PSRCP to steroid rechallenge with survival to discharge.

**Conclusions**: As COVID-19 becomes endemic, clinicians should remain wary of the risk of PSRCP. Greater recognition of the importance of steroid weans and rechallenges in C19P narratives will help avoid poor outcomes, readmissions and the risk of post-C19P sequelae. Awareness of the PSRCP phenomenon should lower the threshold for slow steroid weans upon an initial C19P diagnosis over the standard UK regimen of a 10-day duration or less dexamethasone course. A definition for PSRCP is proposed as well as a decision aid around steroid strategies in patients both with and at risk of PSRCP.

**Supplementary Information:**

The online version contains supplementary material available at 10.1186/s12890-025-03749-z.

## Background

As the COVID-19 pandemic wanes and the world becomes less immunologically SARS-CoV-2 naïve due to exposure and vaccination, COVID-19 pneumonitis (C19P) is becoming an increasingly less common diagnosis [1]. This could lead to a reduced awareness of the complexities of C19P when diagnosed in the near future. Following the pioneering RECOVERY trial, the standard of treatment for acutely hypoxic COVID-19 pneumonitics in the UK remains dexamethasone 6 mg once a day or equivalent steroid doses for 10 days or till discharge if sooner, a surrogate for return to normoxia [2].

However, this “one size fits all” approach, although necessary and evidence-based, can run into problems when applied to larger populations. This in part reflects the spectrum of interpersonal variation in the host response to both infection and subsequent interventions. Following early recognition of COVID-19 (C19) as having often a biphasic natural history and of the symptom rebound phenomenon in untreated disease, there are now case series and observational studies of relapsing cases after steroid cessation in severe disease [3–7]. This said, post-antiviral symptom rebound remains the more documented type of post-intervention C19 rebound phenomenon in the literature and wider media described as comprising a relapse of symptoms and/or a return of PCR positivity [8–13]. This balance needs to be redressed given the importance of steroids in the C19P armamentarium.

In our institution, we have encountered hypoxic C19P patients who initially responded well to standard steroid therapy only to show an often profound rebound of symptoms and inflammatory markers upon steroid cessation or wean. This occurred without evidence of new pathology and often with no detected evidence of C19 carriage or reinfection yet retaining marked responsiveness to steroid rechallenge. This case series aims to raise awareness of what we call post-steroid rebound COVID-19 pneumonitis (PSRCP) so that steroids can be restarted or reviewed earlier. We also propose a framework to identify C19P patients at risk of PSRCP and thus likely to need steroid weans and early follow-up. The risk of severe C19P is greater in men, particularly those with cardiovascular disease, diabetes mellitus and obesity [14, 15]. We posited that PSRCP shares these same risk factors.

## Method

PSRCP patients were retrospectively identified from a search through local respiratory department C19P follow-up records between the start of the RECOVERY trial at our institution, March 23rd 2020 and May 31st 2024. All probable PSRCP cases identified underwent rigorous medical notes review to ensure that the inclusion criteria were met. C19 positivity was defined as a positive PCR from nose and throat swabs or sputum. Pneumonitis was confirmed on the index chest x-ray and/or CT by the attending consultant physician, a reporting radiologist and on retrospective review by a respiratory physician. The notes and drug charts were reviewed to confirm that steroids were administered for the indication of a new oxygen requirement due to C19P in accordance with standard UK practice. From this steroid-receiving cohort, definite PSRCP was defined as new hypoxia within 6 weeks of resolution of the initial hypoxia with no evidence of new explanatory significant pathology from the history, radiology and microbiology. At the time of re-presentation, all suspected PSRCP patients underwent a standard screening bundle that included serum beta-d-glucan for those at risk of *pneumocystis* pneumonia, respiratory pathogen throat swab PCR panel testing, clinical exclusion of connective tissue disease when relevant, sputum culture when possible and lastly D-dimer testing and CTPA scans where reasonable concerns of new pulmonary embolism existed. These screening results were retrospectively re-reviewed to ensure robustness of the PSRCP label. Following each confirmation of a definite PSRCP status, data extraction from that case would ensue.

Several inflammatory markers including C-reactive protein (CRP), neutrophil–lymphocyte ratio (NLR), lactate dehydrogenase (LDH) and ferritin were measured during the narratives of some of the patients identified. This was part of an attempt at our institution to better detect C19 hyper-inflammation and thus risk stratify all C19P patients through their journeys given the intrinsic limitations of each marker and the absence of a C19 specific biomarker. However, as CRP and NLR were the only markers consistently checked through both the pre-rebound and rebound narratives of each case, these two readily available and more specific blood tests were chosen as the biomarkers of interest.

## Results

### Demographics

Eighteen patients were identified, 16 of whom were assigned male at birth, 2 assigned female. Among the eighteen identified, 17 were of Caucasian ethnic origin and one was of Southeast Asian origin. This make-up reflects the population mix seen in Worthing and surrounding areas in West Sussex. Sixteen patients in total (89%) had a past or present history of regular smoking. The demographics and baseline characteristics are summarised in Table 1 presented below.

**Table 1:** Demographics and baseline characteristics

Case series size		18 patients
Age range		48–79 years, mean 68
Ethnicity		Southeast Asian 1 (6%), Caucasian 17 (94%)
Biological sex		Female 2 (11%), male 16 (89%)
Year of initial COVID-19 pneumonitis presentation	2021	15 cases (83%)
	2022	2 cases (11%)
	2023	1 case (6%)
Smoking status	Never smoker	2 patients (11%)
	Ex-smoker 1–5 pack-years*	3 patients (17%)
	Ex-smoker > 5–10 pack-years	4 patients (22%)
	Ex-smoker > 10–20 pack-years	3 patients (17%)
	Ex-smoker > 20 pack-years	6 patients (33%)
	Current smoker	0 patients (0%)

^*^one pack-year refers to smoking a standard pack of 20 cigarettes a day for a year

### Comorbidities

**Fig. 1:**
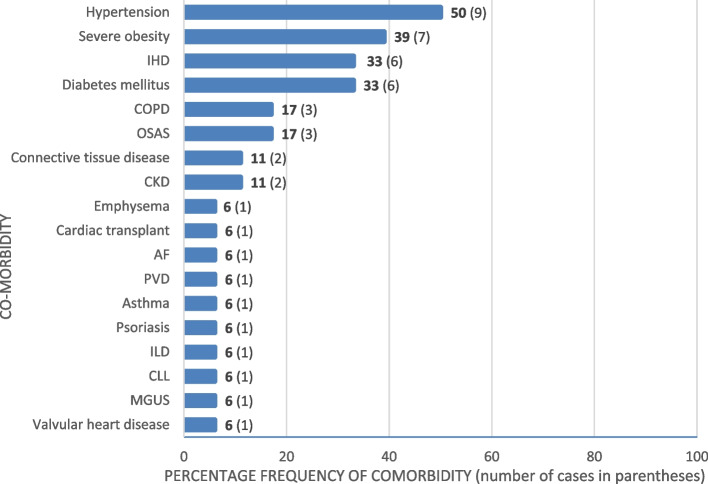
Overall comorbidity breakdown

**Fig. 2:**
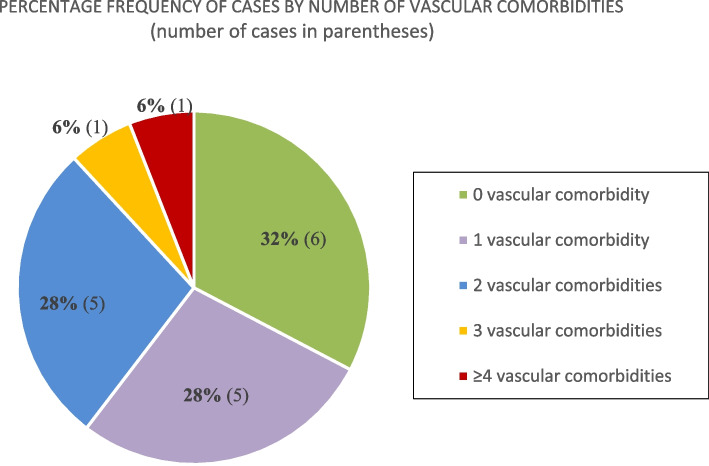
Vascular comorbidity

The most common conditions were hypertension (9, 53%), diabetes mellitus (6,35%), smoking-related pulmonary disease (COPD, emphysema) (5, 29%), ischaemic heart disease (6, 35%) and morbid obesity (7, 41%). Morbid/severe obesity was defined as a BMI ≥ 40 or ≥ 35 with at least one obesity-related health condition [16]. Two of the 18 patients were immunosuppressed with one patient on sirolimus, mycophenolate and prednisolone for heart transplantation and another on rituximab for rheumatoid arthritis. The comorbidity landscape is summarised in Figs. 1 and 2 below.

Details of the initial admission narratives for the cohort are detailed in Table 2.

**Table 2:** Index COVID-19 pneumonitis narrative and vaccination status breakdown

				Number	Percentage
Vaccination status		Fully vaccinated or boosted > 6 mths prior to index illness symptom onset		1	5.5
		Fully vaccinated or boosted 3-6 mths prior to index illness symptom onset		7	39
		Fully vaccinated or boosted < 3 mths prior to index illness symptom onset		0	0
		Partly vaccinated pre-index illness symptom onset		1	5.5
		Unvaccinated		9	50
Admission	*Index observations*	Respiratory failure? (pa0_2_ < 8.0 kPa)		18	100
		Fever		3	17
	*Initial CXR findings*	Pneumonitic changes?		18	100
	*Index inflammatory biomarkers*	Presence of lymphopaenia? (< 1.0 × 10^9^/l)		11	61
		N-L ratio (normal range 0.8–3.5)	Normal	3	17
			> 3.5–6.9	7	39
			≥ 7	8	44
		CRP (mg/dl)/normal range 0–5	0–5	0	0
			≥ 5–74	1	6
			> 74–99	4	22
			≥ 100–200	10	55.5
			> 200	3	16.5
Management	*Pharmacological*	Supportive		0	0
		Steroids	Standard course to discharge/normoxia	15	83
			Weaning regimen	3	17
		Remdesivir		0	0
		IL-6 inhibitor		6	33
		Baricitinib		3	17
	*Maximum oxygen support required*	Facemask oxygen		15	83
		CPAP		3	17
		Invasive ventilation		0	0
Outcome pre-rebound		% weaned off oxygen pre-rebound		16	89
		Time to oxygen wean	< 14 days	10	56
			14–27 days	5	28
			≥ 28 days	1	6
		% discharged at time prior to rebound		16	89

The narratives of the cohort from PSRCP diagnosis to discharge are summarised in Tables 3 and 4.

**Table 3:** Steroid landscape and clinical status at index PSRCP presentation with subsequent management plan

				Number	Percentage
"Index PSRCP background atpresentation (cases 18)	*On steroids at time of initial rebound?*	Longterm steroid for non-C19P indication		2	11
		Weaning regimen for C19P indication		3	17
	*Blood test results ≤48 h pre-index C19P steroid wean / cessation or hospital discharge*	Lymphopaenia? (< 1.0 × 10^9^/l)		4	27
		CRP (mg/dl)/normal range 0–5	0–5	4	27
			> 5–74	10	66.5
			> 74–99	0	0
			> 99	1	6.5
	*Duration from C19P steroid cessation to initial PSRCP symptoms*	< 7 days		6	40
		7–14 days		4	27
		> 14–28 days		2	13
		> 28 days		3	20
	*PCR (17 cases re-tested)*	Still positive?		5	33
	*Index observation*	Fever?		4	22
		Respiratory failure? (pa0_2_ < 8.0 kPa)		16	89
	*Inflammatory markers at presentation*	Lymphopaenia? (< 1.0 × 10^9^/l)		5	28
		NLR ratio (normal range 0.8–3.5)	Normal	5	28
			> 3.5–6.9	5	28
			≥ 7	8	44
		CRP (mg/dl)/normal range 0–5	0–5	0	0
			> 5–74	7	39
			75–99	1	6
			≥ 100–200	6	33
			> 200	4	22
Management strategy	*Initial steroid strategy*	Dexamethasone 6 mg		7	39
		Equivalent dose prednisolone		9	50
		IV methylprednisolone and PO stepdown		2	11
	*Total steroid strategy for index rebounds and relapse rate*	≤ 10 day course of high dose oral steroid	Relapsed?	2	100
			No relapse	0	0
		Slow steroid wean post initial high oral dose	Relapsed?	6	43
			No relapse	8	57
		IV methylprednisolone and PO steroid wean	Relapsed?	0	0
			No relapse?	2	100

**Table 4:** Outcomes of patients with PSRCP (as of June 2024)

			Number	Percentage
Total number of post-steroid rebounds per patient	3 rebounds		2	11
	2 rebounds		6	33
	1 rebound		10	56
Steroid wean success defined as cessation of steroids commenced for C19P therapy for ≥ 6 weeks	Successful wean	< 6 months	4	22
		> 6 months	7	39
		Unknown	1	5.5
	Still on C19P steroids		3	17
	Died on C19P steroids		2	11
	Continuing steroid for non-C19P indication		1	5.5
Survival	To discharge post-index rebound		17	94
	1 year survival *(17 diagnosed* ≥ *1 year prior)*		13	76
Fibrotic changes radiologically confirmed post-rebound?			5	28

### Who rebounds off steroids?

Sixteen (89%) of the 18 rebounders were male and 16 in total of 18 (89%) patients had a cigarette smoking history. Hypertension (9/18), diabetes mellitus (6/18), severe obesity (7/18) and IHD (6/18) seemed to be major predictors of PSRCP. A total of 9/18 patients (50%) were unvaccinated but this and the noted comorbidities are well recognised themselves as predictors of COVID-19 pneumonitis [11, 12]. However there were also trends in the index narratives. The majority of these patients had lymphopaenia, 11/18 (61%) or a raised neutrophil–lymphocyte ratio, 15/18 (83%) and all bar one had a CRP level ≥ 75 mg/dl. Although 5 of 18 C19P patients were on ongoing steroids at the time of PSCRP symptom onset, only 3 were weaning regimens as 2 were on long-term steroids for non-COVID-19 indications. As such, 83% (15/18) of PSRCP patients were individuals treated with a short course of dexamethasone as opposed to a weaning regimen. Fifteen of these patients had a CRP test within 48 h or less from the date of steroid cessation, steroid wean or discharge and of those, 11 (73%) still had an elevated CRP.

### When do patients rebound off steroids?

Two patients rebounded in hospital while 16 did so after discharge. The time from steroid cessation to rebound in the cohort of all patients given a short course of dexamethasone at initial presentation including those receiving long-term low dose steroid and/or pre-existing immunosuppression (15 individuals) ranged from 1 to 40 days. Twelve of this stated sub-cohort of 15 patients (80%) developed symptoms within 28 days of steroid cessation.

### How do you know they are rebounding?

The rates of CRP elevation and raised neutrophil–lymphocyte ratios in PSRCP presentations were not dissimilar to rates in the index C19P presentations. All 18 patients had an elevated CRP at index C19P presentation and at initial PSRCP presentation. Fifteen of the 18 (83%) had raised NLR ratios at index C19P presentation in comparison to 13 of 18 (72%) at first PSRCP presentation. However, lymphopaenia seemed to be less common in PSRCP presentations at 4 of 18 (22%) vs 15 of 18 (83%) index C19P presentations. If the steroid-free at time of PSRCP are considered, lymphopaenia remains less common than in the index C19P cohort at 3/13 patients (23%) vs 11/18 patients (61%). PCR status in the PSRCP cohort was not predictive in of itself as there was a high rate of PCR status conversion from 100% positive at C19P presentation to 24% (4 patients) positive in the 17 re-tested.

### Who relapses after an initial PSRCP episode?

As summarised in Table 4, there were two rebounders managed with short courses of dexamethasone and both had subsequent rebounds whereas the two who had IV methylprednisolone and a steroid wean thereafter did not rebound again. In the middle, 42% (6/14) of the cohort managed with dexamethasone or prednisolone re-challenges and slow steroid weans thereafter had at least one further relapse. 

### Prognosis and prognostic markers

Survival to discharge following the index rebound was 94% representing 17 of the 18 patients whereas the 3-month survival was 16 out of 18 (89%). The 12-month survival of the 17 rebounds that occurred over 1 year from time of initial data capture was 13/17 (76%). Five of the 18 patients in total (28%) demonstrated fibrotic changes on CT post index COVID-19 pneumonitis in the form of parenchymal distortion with traction bronchial dilation or honeycombing.

## Discussion and conclusions

This case series is the first to explore the concept of PSRCP in a UK population. Broadly speaking a similar phenomenon could also occur with other COVID-19 manifestations such as gastrointestinal and cardiac presentations and the whole process is almost certainly an analogue of the biphasic presentations seen at the start of the pandemic as well as post-antiviral rebound narratives [17, 18]. Notably, former United States president, Joe Biden has experience of a post-nirmatrelvir-ritonavir (Paxlovid™) rebound [19]. One could extrapolate this notion of a smouldering autoimmune process to underpin the aetiology of “long COVID” or more specifically the post-COVID-19 syndrome [20, 21].

As illustrated by the phases of disease concept at the start of the pandemic with coryzal, consolidatory and then pneumonitic phases, C19 end organ disease has a large autoinflammatory component [7, 22]. C19P is clinically, radiologically and histologically an accelerated form of (post-)viral fibrotic organising pneumonia, a phenomenon well known in respiratory medicine. In these contexts, it follows that in theory inflammation can persist despite intervention when the following conditions are met:

1. Delayed viral clearance potentially from reasons of impaired structural or humoral host defence (such as immunosuppression/compromise) or viral naïveté. This readily explains viral PCR swab positivity rebounds following use of antivirals.
2. An exaggerated and prolonged initial inflammatory response even in the face of probable viral clearance due to the presence of either:
  i. Pre-existing disordered autoimmunity such as connective tissue disease
  ii. Pre-existing pro-inflammatory substrate as seen in microvascular diseases such as diabetes, peripheral and cardiac vascular disease and metabolic syndromes leading to persistent and profound auto-inflammation.

In either of these situations, there will be an increased risk of symptom recurrence on cessation of immunomodulation for acute severe C19P irrespective of viral swab PCR status, itself a test with limitations. Simply put, steroids merely aim to suppress with varying levels of success an inflammatory cascade and cytokine storm that can have a protracted life span. The term “rebound” implies springing back and more accurately captures the acuity and severity of the phenomenon in question and its occurrence after short-term steroid use than a word such as “relapse”. It is these qualities of acuity and severity that differentiate PSRCP from relapse events that can follow steroid wean or withdrawal in steroid-responsive diseases such as asthma and connective tissue diseases.

This case series highlights a sequela of COVID-19 that is still widely unrecognised especially when repeated viral swabs are negative and often leads to over-investigation and delayed immunomodulation. This can increase length of stay and increase the risk of post-COVID-19 fibrotic disease, pulmonary emboli and pneumothorax/pneumomediastinum by virtue of continuing hyperinflammation [23–25]. The term COVID-19 rebound is often used to encompass both a viral PCR status relapse as well as a symptom rebound and the diagnosis is often linked arguably too tightly to a positive PCR status. The limitations of throat swab PCR positivity for COVID-19 diagnostics are well recognised. We propose a definition for PSRCP that is more specific and holds more relevance for front-door acute clinicians while also encompassing the more smouldering, swab PCR negative subtype of this phenomenon sometimes called post-COVID-19 pneumonitis in the literature [24–27].

Accepting the limitations of the cohort size which reduces generalisability, the five proposed criteria for a definite post-steroid rebound COVID-19 pneumonitis (PSRCP) diagnosis are as follows:

**Table:** 

1. Evidence of positive clinical response to immunomodulation for index C19P presentation
2. Recurrence or relapse of the presenting symptoms of C19P
3. It should occur within 6 weeks of the cessation of or reduction in immunomodulation to which there was positive response
4. There must be biochemical and radiological evidence of ongoing inflammation
5. There must be, as much as possible, exclusion of new causes of inflammation including non-COVID-19 infection, thrombotic or embolic events and autoimmune diseases.

This report also adds to a body of knowledge that suggests the one size fits all steroid approach to COVID-19 pneumonitis as advised by NHS England for understandable simplicity has drawbacks [28]. This case series, limited In size as it is, does also suggest co-morbidities that are possible adverse outcome predictors C19 clinicians should be aware of. These may steer C19P care towards slow steroid weans at index presentation and at rebound. These predictors are diabetes, hypertension, ischaemic heart disease, severe obesity as well as a persisting elevated CRP test, findings that correlate with previous work [4, 5].

Currently at our institution, first presentation cases of C19P with any of these four comorbidities but with normal inflammatory markers at discharge are followed up with a clinical review and a reassessment of inflammatory markers including full blood count, CRP, ferritin and LDH at the 2 week, 4 week and potentially 6 week point post-discharge. In parallel, on a case-by-case basis, a short wean of steroids over 4–6 weeks is also considered in this cohort particularly if two or more of these co-morbidities are present. All of the pneumonitics with a persisting CRP elevation at the point of discharge or steroid cessation are considered for a slow wean of steroids over a minimum of 6 weeks. The Worthing weaning approach involves the conversion of dexamethasone 6 mg once daily to an equivalent anti-inflammatory dose of prednisolone 40 mg once daily for ease of weaning. The standard 6-week weaning regimen consists of a reduction every week to 30 mg, followed by 20 mg, 15 mg, 10 mg and 5 mg once daily with regular clinical review monitoring for signs of relapse. A full inflammatory panel blood check alongside a CT scan would follow at the 6-week mark to guide decision-making regarding cessation of low dose steroids or potential continuation/continuing wean till to the 12-week point. This approach is also used in PSRCP with one difference. Upon diagnosis of PSRCP in our institution, a steroid re-challenge proceeds, the dose of which would depend on severity of the episode and current dose of steroids but for those not currently on steroids, this would be at least 0.5 mg/kg of prednisolone and no lower than 40 mg. We chose to mirror current UK guidance on use of dexamethasone in initial presentations of C19P in hypoxic respiratory failure and thus continue the steroid re-challenge until discharge or 10 days at which point we embark upon a slow wean. However, we favour pausing weaning at a minimum of 10 mg by the 6-week mark. At this juncture, standard 6-week reassessment would proceed with a view to potentially completing a steroid wean over a 12-week total period minimum. A decision aid flow chart around steroid wean decision-making is included in Supplementary Information under the heading Supplementary Material 1.

All this said, it may not be possible at the front door to confidently exclude co-existing or contributing de novo infection; thus a broad approach may be required alongside, when applicable, input from a multidisciplinary forum comprising respiratory physicians, radiologists and microbiologists to diagnose and manage PSRCP.

The protective response to vaccinations has a time limit while provoking a suboptimal response in many individuals, particularly immunocompromised individuals and there are still, even at this stage of the pandemic, a significant number of persons who remain C19 naïve [29]. PSRCP is thus a phenomenon that is here to stay for the foreseeable future in all likelihood. More robust and larger datasets are needed to explore this fully and to guide research in this area. A key focus would be the development, in lieu of a C19-specific biomarker, of a prediction score using parameters such as inflammatory markers, (micro)vascular disease burden, smoking history, sex and age to create a means of predicting who is likely to rebound and who should thus be kept under close surveillance with a slow steroid wean. There are further questions regarding what the ideal steroid weaning stratagem should be in terms of dose and duration and whether there is a role for early institution of steroid-sparing immunosuppressants. Drugs such as mycophenolate mofetil are often used in acute/subacute inflammatory interstitial lung diseases to avoid the side-effects of prolonged steroid use.

## Supplementary Information

Supplementary Material 1.

Supplementary Material 2.

## Data Availability

All data analysed during this study is included in this published article and its supplementary information files.

## Abbreviations

AF: Atrial fibrillation
BMI: Body-mass index
C19: COVID-19
C19P: COVID-19 pneumonitis
CKD: Chronic kidney disease
CLL: Chronic lymphocytic leukaemia
COPD: Chronic obstructive pulmonary disease
CPAP: Continuous positive airway pressure
CRP: C-reactive protein
CT: Computed tomography
CTPA: Computed tomography pulmonary angiogram
FBC: Full blood count
h: Hours
IHD: Ischaemic heart disease
IL6: Interleukin 6
ILD: Interstitial lung disease
IV: Intravenous
kg: Kilogrammes
LDH: Lactate deydrogenase
mg: Milligrammes
MGUS: Monoclonal gammopathy of unknown significance
PCR: Polymerase chain reaction
PO: *per os* (Oral)
PSRCP: Post-steroid rebound COVID-19 pneumonitis
PVD: Peripheral vascular disease
