# Supplementary material for: Post-steroid rebound in COVID-19 pneumonitis: a case series and review of the literature
Source: BMC Pulm Med. 2025 Sep 30;25:440. doi: 10.1186/s12890-025-03749-z (PMC12487355; doi:10.1186/s12890-025-03749-z)
Supplement: Supplementary file 1 — Supplementary Material 1. [file 12890_2025_3749_MOESM1_ESM.docx]

| **COVID-19 PNEUMONITIS INPATIENT AT POINT OF STEROID CESSATION**  **(discharge or D10 of standard / rechallenge steroid therapy)** |
| --- |
| Check CRP  Review steroid tolerance  Ensure BM control |

**STEROID WEAN IN COVID-19 PNEUMONITIS**

| Index OR Rebound? |
| --- |

| **INDEX COVID—19 PNEUMONITIS PRESENTATION** |
| --- |

| **POST-STEROID REBOUND COVID-19 PNEUMONITIS PRESENTATION** |
| --- |

| Elevated CRP with no alternative aetiology? |
| --- |

| No |
| --- |

| Yes |
| --- |

| ADVISE STEROID WEAN |
| --- |
| Consider baseline LDH and ferritin to aid monitoring |

| High PSRCP risk comorbidity? |
| --- |

| High PRSCP risk comorbidity? |
| --- |

No

Yes

| No |
| --- |

|  |
| --- |

| NOT FOR STEROID WEAN.  FOR STANDARD 12-WEEK FOLLOW-UP |
| --- |

| CONSIDER STEROID WEAN (4-6 weeks)  **OR**  REVIEW AT 2, 4 AND 6 WEEKS |
| --- |

| PROCEED WITH STEROID WEAN (4-6 weeks)  WITH REVIEW AT 2,4 AND 6 WEEKS |
| --- |

| PROCEED WITH STEROID WEAN  (6 weeks minimum) WITH REVIEW AT  2,4 AND 6 WEEKS |
| --- |

| PROCEED WITH SLOW STEROID WEAN  (12 weeks minimum) WITH REVIEW AT 2,4, 6 AND 12 WEEKS |
| --- |

Yes

Yes
