# Supplementary material for: Post-steroid rebound in COVID-19 pneumonitis: a case series and review of the literature
Source: BMC Pulm Med. 2025 Sep 30;25:440. doi: 10.1186/s12890-025-03749-z (PMC12487355; doi:10.1186/s12890-025-03749-z)
Supplement: Supplementary file 2 — Supplementary Material 2. [file 12890_2025_3749_MOESM2_ESM.docx]

|  | Age at  first C19 event | M/F | Index event admission year | Definite  fever? (≥38^O^C)  Y / N | Initial event  C19-specific strategy | Level of support needed | Blood results at presentation | | | Time to sats ≥ 92%  (days) | Past medical history | | | | | Bloods ≤48hrs prior to discharge | | No. of days off C-19 steroids till rebound symptoms | Pre-morbid vaccine history and timing from symptom onset | | | |
| --- | --- | --- | --- | --- | --- | --- | --- | --- | --- | --- | --- | --- | --- | --- | --- | --- | --- | --- | --- | --- | --- | --- |
|  |  |  |  |  |  |  | CRP  mg/l | Lym  10^9^/l | Neu  10^9^/l |  | Resp | CV | Endo | BMI  > 35 | Other | CRP | Lym |  | Any dose at all? Y/N | 1^st^ dose and timing / mths | 2^nd^ dose and timing / mths | Boost dose and timing / mths |
| 1 | 66 | m | 2021 | n | Dex | CPAP | 144 | 1.3 | 4.8 | 42 | Ex-skr | HTN | Nil | N | MGUS | 31 | 1.3 | 40 | N | n/a | | |
| 2 | 71 | m | 2021 | n | Dex | VM | 38 | 0.9 | 5.3 | 11 | Ex-skr | HTN | DM | y | nil | 3 | 1.0 | 27 | N | n/a | | |
| 3 | 74 | m | 2021 | n | Dex | VM | 132 | 1.0 | 14.6 | 15 | Ex-skr, COPD | HTN, CKD | Nil | n | nil | 81 | 0.6 | 3 | N | n/a | | |
| 4 | 67 | m | 2021 | n | Dex | NC | 265 | 1.2 | 8.5 | n/a | Ex-skr | IHD, HTN, PVD, CKD | Nil | n | psoriasis | 46 | 1.0 | 11 | N | n/a | | |
| 5 | 64 | m | 2021 | y | Dex; pred wean | NRB | 110 | 0.9 | 5.4 | 15 | OSAS | HTN | DM | Y | PE | 15 | 1.8 | n/a*^6^ | N | n/a | | |
| 6 | 77 | m | 2021 | n | Dex | VM | 305 | 0.6 | 3.7 | 14 | Ex-skr, e’sema | AF | Nil | Y | nil | 62 | 1.0 | 37 | N | n/a | | |
| 7 | 64 | F | 2021 | n | Dex | VM | 144 | 0.8 | 10.5 | 9 | Ex-skr | nil | Nil | n | nil | nd | nd | 17 | N | n/a | | |
| 8 | 77 | m | 2021 | n | Dex; S’mab | CPAP | 208 | 0.9 | 6.2 | n/a (2l) *^1^ | Ex-skr | HTN | Nil | y | nil | dex stop day*^4^ | | 5 | Y | Y, 8 | Y, 6 | N |
|  |  |  |  |  |  |  |  |  |  |  |  |  |  |  |  | 2 | 1.3 |  |  |  |  |  |
| 9 | 68 | m | 2021 | n | Dex; S’mab | VM | 81 | 3.8 | 7.5 | 4 | Ex-skr | IHD, HTN | Nil | n | CLL | nd | nd | 11 | Y | Y, 7 | Y, 5 | N |
| 10 | 69 | m | 2021 | n | Dex | VM | 81 | 0.9 | 2.2 | 10 | Ex-skr / OSAS | IHD, AS, HTN | DM | y | nil | 4 | 1.8 | 9 | N | n/a | | |
| 11 | 70 | m | 2021 | n | Dex; S’mab; pred wean | NRB | 193 | 0.8 | 9.9 | 25 | Ex-skr /COPD/ OSAS | IHD, HTN | DM | y | PVD | nd | nd | n/a*^7^ | Y | Y, 8 | Y, 6 | N |
| 12 | 51 | m | 2021 | n | Dex; B’nib; pred wean | CPAP | 99 | 1.2 | 11.6 | 20 | Ex-skr | HTN | DM | y | nil | 13 | 1.7 | n/a*^8^ | Y | Y, 1 | N | N |
| 13 | 48 | m | 2021 | n | Dex; S’mab; B’nib | VM | 97 | 0.7 | 3.8 | 2 | Ex-skr | nil | Nil | n | Seroneg RA | nd | nd | 1 | N | n/a | | |
| 14 | 62 | m | 2022 | y | Dex; T’mab | VM | 118 | 0.6 | 10.4 | 6 | Ex-skr | IHD, CKD, cardiac t’plant | DM | n | CKD | 47 | 1.1 | 4 (long-term pred 10mg) *^9^ | Y | Y, 14 | Y, 12 | Y, 5 |
| 15 | 73 | m | 2021 | y | Dex; S’mab | VM | 110 | 1.0 | 5.9 | 1 | Ex-skr, COPD | nil | nil | y | IA | 60 | 1.4 | 4 ^*10^ | Y | Y, 8 | Y, 6 | N |
| 16 | 71 | m | 2022 | n | Dex | VM | 133 | 0.5 | 7.6 | 8 | Ex-skr  ILD | HTN, IHD | nil | n | nil | 2 | 0.7 | 11 | Y | Y, 22 | Y, 20 | Y, 13 |
| 17 | 79 | f | 2022 | n | Dex; B’nib | VM | 185 | 1.7 | 3.8 | n/a *^2^ (1l) | Asthma, ex-skr, e’sema | Nil | nil | n | RA / on rituximab *^3^ | dex stop day*^5^ | | 3 | Y | Y, 21 | Y, 19 | Y, 3 |
|  |  |  |  |  |  |  |  |  |  |  |  |  |  |  |  | 111 | 0.8 |  |  |  |  |  |
| 18 | 76 | m | 2023 | n | Dex | VM | 199 | 0.4 | 8.8 | 10 | Nil | Nil | Nil | n | nil | 22 | 0.9 | 33 | Y | Y, 34 | Y, 32 | Y, 3 |

**SUPPLEMENTARY DATA TABLES**

|  | Steroid type and current dose at time of rebound | C19 PCR status at rebound | Steroid choice and dose given at rebound | No. of days taken till normoxia post steroid rechallenge | Blood landscape pre-steroid rechallenge at rebound | | | | | Fever at rebound? | Inpatient rebound  outcome | Steroid  plan at d/c | No. of further rebounds | Survived to d/c?  Y/N | Survival in months from rebound diagnosis as of 17/6/24 | Steroid wean narrative from rebound diagnosis as of 17/6/24 | | Complications of note during index and rebound narrative |
| --- | --- | --- | --- | --- | --- | --- | --- | --- | --- | --- | --- | --- | --- | --- | --- | --- | --- | --- |
|  |  |  |  |  | LDH iu/l | CRP  mg/l | Fer  μg/l | Lym  10^9^/l | Neu  10^9^/l |  |  |  |  |  |  | Weaned off C19 steroid? Yes ***or*** No and current dose | Time on C19 steroids / months |  |
| 1 | 0 | neg | Pred 40mg | 0 | 282 | 80 | 775 | 3.1 | 10.5 | N | D/c | Pred wean | 2 | Y | 38 | 7.5 | 37*^16^ | Post-C19 fibrotic ILD, pneumo-mediastinum |
| 2 | 0 | neg | Dex 6mg | 0 | 472 | 7 | 297 | 1.7 | 6.8 | N | D/c | Dex  wean | 2 | Y | 1 (died) | n/a ^*12^ | n/a | Pneumo-mediastinum |
| 3 | 0 | neg | Dex 6mg | 1 | ND | 141 | ND | 1.1 | 12.8 | N | D/c | Nil steroids | 1 | Y | 41 | n/a^*13^ | n/a | nil |
| 4 | 0 | pos | Pred 30mg | 13 | ND | 277 | ND | 0.9 | 7.3 | Y | D/c | Pred wean | 0 | Y | 9 (died) | Y | 7 | nil |
| 5 | Pred 15mg | neg | Dex 6mg (+ t’mab) | 13 | ND | 306 | ND | 1.2 | 13.9 | y | d/c | Pred 40mg and wean | 0 | Y | 39 | Y | 4 | nil |
| 6 | 0 | neg | Pred 40mg | 17 | ND | 48 | ND | 1.3 | 4.1 | N | D/C | Pred wean | 0 | Y | 21 (died) | y | 3 | Post-C19 fibrotic ILD |
| 7 | 0 | neg | Dex 6mg | 3 | ND | 185 | ND | 1.5 | 6.7 | y | d/c | Pred 40mg and wean | 1 | Y | 40 | Y | 15*^17^ | Post-C19 fibrotic ILD |
| 8 | 0 | neg | Pred 40mg | 7 | ND | 32 | ND | 2 | 7 | N | D/c | Pred wean | 0 | Y | 31 | Y | 38 | nil |
| 9 | 0 | neg | MTP 1g | n/a *^10^ | ND | 2 | 907 | 2.7 | 5.9 | N | d/c | Pred 50mg and wean | 0 | Y | 15 | Y | 13 | Post- C19 fibrotic ILD |
| 10 | 0 | neg | Pred 40mg | 5 | ND | 108 | ND | 2.2 | 3.3 | N | d/c | Pred wean | 0 | Y | 39 | Y | 3 | nil |
| 11 | Pred 5mg | neg | Pred 30mg | 9 | 285 | 146 | 320 | 1.3 | 11 | n | d/c | Pred wean | 0 | Y | 31 | Y | 13 | PE |
| 12 | Pred 15mg | neg | Pred 60mg | ***5*** | 324 | 199 | 258 | 1.1 | 5.8 | n | dc | Pred wean | 0 | Y | 30 | Y | 8 | nil |
| 13 | 0 | pos | Dex 6mg +  ronapreve | 4 | ND | 8 | ND | 1.1 | 7.1 | n | d/c | Nil steroids | 1 | Y | 30 | n/a^*14^ | n/a | Pneumo-thorax |
| 14 | Pred 10mg (longterm) | pos | Dex 6mg | 3 | ND | 175 | ND | 0.8 | 20.4 | y | Died | Pred 40mg wean | 1 | N | 1 (died) | n/a *^15^ | n/a | nil |
| 15 | Pred 12.5mg  (longterm) | pos | Dex 6mg | 2 | ND | 6 | ND | 1.1 | 9.9 | y | d/c | Pred 30mg wean | 1 | Y | 32 | Y | 7*^18^ | nil |
| 16 | 0mg | pos | MTP 500mg | 11 *^11^ | 333 | 202 | 1271 | 0.6 | 8.1 | n | d/c | Pred 40mg wean | 0 | Y | 17 | 20 | 17 | nil |
| 17 | 0mg | Not done | Pred 60mg | 9 | ND | 201 | 499 | 0.9 | 7.1 | n | d/c | Pred wean | 1 | Y | 18 | Y | unk*^19^ | nil |
| 18 | 0mg | Neg | Pred 30mg | 1 | 277 | 33 | 208 | 1.5 | 4.7 | n | d/c | Pred wean | 0 | Y | 4 | 5 | 4 | Post-c19 fibrotic ILD, PE, DVT |

**NOTES**

**Abbreviations**

^O^C: Degrees Celsius

μg: microgrammes

AF: atrial fibrillation

AS: Aortic stenosis

b’nib: Baricitinib

BMI: Body mass index

C-19: Covid-19

CKD: Chronic kidney disease

CLL: Chronic lymphocytic leukaemia

COPD: Chronic obstructive pulmonary disease

CPAP: Continuous positive airway pressure

CRP: C-reactive protein

CV: Cardiovascular and vascular diseases

D/c: Discharge

Dex: Dexamethasone

DM: Diabetes mellitus

DVT: Deep vein thrombosis

E’sema: Emphysema

Endo: Endocrinological

Ex-skr: Ex-smoker

F: Female

Fer: Ferritin

g: gramme

hrs: hours

HTN: Hypertension

IA: Inflammatory arthritis

IHD: Ischaemic heart disease

ILD: Interstitial lung disease

l: litre

LDH: Lactate dehydrogenase

Lym: Lymphocytes

M: Male

Mg: milligrammes

MGUS: monoclonal gammopathy of unknown significance

mths: Months

MTP: intravenous Methylprednisolone

N/A: Not applicable

NC: Nasal cannula

ND: Not done

Neu: Neutrophils

Neg: Negative

No.: Number

NRB: non-rebreathe mask

OSAS: Obstructive sleep apnoea syndrome

PCR: Polymerase chain reaction

PE: Pulmonary emboli

Pos: Positive

PVD: Peripheral vascular disease

Pred: Prednisolone

RA: Rheumatoid arthritis

Resp: Respiratory

Sats: Saturations

Seroneg: Seronegative

S’mab: Sotrovumab

T’mab: Tocilizumab

T’plant: Transplant

Unk: Unknown

VM: Venturi mask

**NB.**

*^1, 2^ Case 8 and 17 rebounded as inpatients while still on low dose nasal cannula oxygen and did not achieve complete wean at end of index episode. Nadir

oxygen requirement recorded in parenthesis in column “Time to saturation ≥ 92% (days)”.

*^3^  Case 17 was a patient with seropositive rheumatoid arthritis on long term regular rituximab

*^4, 5^ Case 8 and 17 were still inpatients at time of steroid cessation and subsequent rebound and as such, what is recorded in the “Bloods ≤48hrs pre dc or

rebound” column are values within 48 hrs prior to day of inpatient steroid cessation. Values of other patients in this column represent blood tests

done within 48 hours prior to discharge which aligned with timing of steroid cessation in all 16 patients

*^6,7,8^ Cases 5, 11 and 12 were still on reducing doses steroids in the form of a prednisolone wean after the primary standard course of dexamethasone at

the onset of rebound symptoms

*^9^  Case 15 was on long term prednisolone with MMF and sirolimus for a cardiac transplant. This patient returned to this dose after a standard course of

dexamethasone for Covid-19 pneumonitis at index presentation. Number of days till rebound symptoms on baseline prednisolone dose after

dexamethasone cessation is presented.

*^10^ Case 9 did not achieve normoxia and required long term oxygen supplementation post rebound

*^11^ Case 15 was on 1l nasal speculum prior to the rebound and thus the quoted result of 11 days refers to a return to this baseline rather than normoxia

*^12^  Case 2 died prior to steroid wean commencement due to complications from pneumomediastinum and pneumonia

*^13^ Case 3 had 1 day of dexamethasone at initial rebound due to failure of diagnosis. A prednisolone wean was commenced after second rebound which

was weaned off after 2 months.

*^14^  Case 13 had 5 days of dexamethasone at initial rebound. A prednisolone wean was commenced after second rebound which was weaned off after 2

months.

*^15^ Case 14 died as an inpatient during his rebound admission and as such the steroid plan listed refers to the approach during discharge planning.

Steroid wean was paused at 25mg of prednisolone due to second rebound and death.

*^16^ Case 1 rebounded a second time after 1 week off prednisolone after initial 1 month prolonged course. Rebounded a third time after 2 weeks post

completion of slow 3 month wean off prednisolone from 40mg to zero. Following subsequent very slow wean on steroids, remains on 7.5mg of

steroids. Total time on steroids is 37 months as of June 2024.

*^17^ Case 7 rebounded a second time after prednisolone wean from 30mg to 20mg over 3 weeks. Patient was given tocilizumab and rechallenged with via

weaning regimen of dexamethasone from 6mg till clinical stability; ultimately was weaned off equivalent dose of prednisolone slowly. Total time on

steroids was 15 months.

*^18^ Case 15 rebounded a second time after prednisolone wean from 30mg to 5mg over 3 weeks. Prednisolone wean re-commenced at slower rate over a

total of 6 months to a baseline of 5mg for his pre-morbid inflammatory arthritis. Thus total time on C-19 steroids is 7 months.

*^19^ Case 17 post-rebound care was lost to follow-up
